# Short-Term Effects of Visceral Manual Therapy on Autonomic Nervous System Modulation in Individuals with Clinically Based Bruxism: A Randomized Controlled Trial

**DOI:** 10.3390/dj13070325

**Published:** 2025-07-16

**Authors:** Cayetano Navarro-Rico, Hermann Fricke-Comellas, Alberto M. Heredia-Rizo, Juan Antonio Díaz-Mancha, Adolfo Rosado-Portillo, Lourdes M. Fernández-Seguín

**Affiliations:** 1 Department of Physiotherapy, Faculty of Nursing, Physiotherapy and Podiatry, Universidad de Sevilla, 41009 Sevilla, Spain; caynavric@alum.us.es (C.N.-R.); jdm@us.es (J.A.D.-M.); adorospor@alum.us.es (A.R.-P.); lfdez@us.es (L.M.F.-S.); 2 Instituto de Biomedicina de Sevilla—IBiS, Hospitales Universitarios Virgen del Rocío y Macarena/Consejo Superior de Investigaciones Científicas (CSIC)/Universidad de Sevilla, 41013 Sevilla, Spain; 3 Understanding Movement and Self in Health from Science (UMSS) Research Group, 41009 Andalusia, Spain

**Keywords:** bruxism, autonomic nervous system, physical therapy modalities, diaphragm, musculoskeletal manipulations

## Abstract

**Background/Objectives**: Bruxism has been associated with dysregulation of the autonomic nervous system (ANS). Visceral manual therapy (VMT) has shown beneficial effects on the vagal tone and modulation of ANS activity. This study aimed to evaluate the immediate and short-term effects of VMT in individuals with clinically based bruxism. **Methods**: A single-blind randomized controlled trial was conducted including 24 individuals with clinically based bruxism. Participants received two sessions of either VMT or a sham placebo technique. Outcome measures included heart rate variability (HRV), both normal-to-normal intervals (HRV-SDNN), and the root mean square of successive normal-to-normal intervals (HRV-RMSSD), as well as muscle tone and stiffness and pressure pain thresholds (PPTs). Measurements were made at T1 (baseline), T2 (post-first intervention), T3 (pre-second intervention), T4 (post-second intervention), and T5 (4-week follow-up). **Results**: A significant time*group interaction was observed for HRV-SDNN (*p* = 0.04, η^2^ = 0.12). No significant changes were found for muscle tone or stiffness. PPTs significantly increased at C4 after the second session (*p* = 0.049, η^2^ = 0.16) and at the left temporalis muscle after the first session (*p* = 0.01, η^2^ = 0.07). **Conclusions**: The findings suggest that two sessions of VMT may lead to significant improvements in HRV-SDNN compared to the placebo, suggesting a modulatory effect on autonomic function. No consistent changes were observed for the viscoelastic properties of the masticatory muscles. Isolated improvements in pressure pain sensitivity were found at C4 and the left temporalis muscle. Further research with larger sample sizes and long-term follow-up is needed to determine the clinical relevance of VMT in the management of signs and symptoms in individuals with bruxism.

## 1. Introduction

Bruxism is defined as a repetitive masticatory muscle activity characterized by clenching or grinding of the teeth and/or by bracing or thrusting of the mandible [1]. In healthy individuals, bruxism is not considered a disorder, but it may exhibit both harmful and potentially protective or adaptive functions, depending on its intensity, frequency, duration, and context [1]. It is also a risk factor for adverse oral health outcomes [2], particularly when the intensity and direction of the clenching forces exceeds the adaptive capacity of the stomatognathic system, potentially leading to TMD muscular and/or articular symptoms [3].

The global prevalence of bruxism (both awake and sleep forms) is estimated at 22% in the general population [4], and the condition is considered to have a multifactorial nature. Contributing etiological factors may include genetics [5], psychosocial aspects (e.g., stress, anxiety), and central nervous system components, including impairments of brain neurotransmitters [6,7,8]. In addition, other systemic and lifestyle-related conditions—such as obstructive sleep apnea syndrome, gastroesophageal reflux, and poor sleep hygiene, among others—have also been proposed as relevant contributors to the pathophysiology of bruxism [9,10]. In some individuals, psychosocial factors such as anxiety or depression may be associated with or contribute to the development of bruxism behaviors [11]. The amygdala plays a key role in processing emotional responses. Under emotional stress, it triggers the release of the excitatory neurotransmitter glutamate [12]. Amygdala activation has been shown to influence mandibular musculature by stimulating the mandibular motor nucleus, thereby increasing the activity of jaw-closing muscles [13,14]. In the mandibular bone, the mechanical stimulation of jaw closure induces the production of the hormone osteocalcin. Osteocalcin is normally carboxylated by the enzyme gamma-carboxylase, thus becoming inactive and limited only to bone-specific functions. However, glutamate inhibits gamma-carboxylase, leading to the release of uncarboxylated osteocalcin into the bloodstream. This uncarboxylated form may bind to Gprc6a receptors located on vagal neurons, inhibiting the synthesis of acetylcholine and reducing nerve conduction velocity. This entire process may result in increased sympathetic activity [12].

Heart rate variability (HRV) is a non-invasive and objective electrophysiological measure often used to assess the stress level, autonomic nervous system (ANS) status, and general health. HRV is defined as the analysis of fluctuations in the intervals between consecutive heartbeats [15] and serves as a proxy for ANS balance by reflecting vagal parasympathetic activity at the cardiac level [16]. Lower HRV suggests sympathetic dominance and amygdala hyperactivation and has been associated with strong negativity bias and impaired regulatory and homeostatic functions. On the contrary, a high HRV reflects increased parasympathetic (vagal) activity and reduced fear responses [15]. Previous evidence indicates that in individuals with bruxism, HRV shows a predominance of sympathetic activity [17]. This sympathetic hyperactivity is likely related to parasympathetic inhibition, which may be mediated by afferent inputs originating from the abdominal viscera and projecting to the vagal sensory nucleus [18,19].

Several conservative treatment approaches have been proposed for managing bruxism, including occlusal splints [20], behavioral therapy [21], pharmacological treatment [22], and physiotherapy. Among physiotherapeutic modalities, manual therapy (MT) has emerged as a useful non-invasive option [23]. MT has demonstrated beneficial effects on HRV, resulting in decreased activity of the sympathetic nervous system, as well as pain reduction [24]. Visceral manual therapy (VMT), a specific form of MT, has proven effective in various clinical conditions, for example, to regulate the lower esophageal sphincter in patients with gastroesophageal reflux disease [25,26]. VMT may help to modulate the ANS activity through visceral stimulation, which can enhance vagal tone and activate viscerosomatic reflexes, thereby promoting parasympathetic dominance [27]. This could be clinically relevant in individuals with bruxism, a population in which autonomic imbalance has been reported [17]. To date, no previous studies have investigated the impact of VMT on ANS modulation in individuals with bruxism or probable bruxism. The present study aimed to address this gap in the literature by examining whether manual interventions targeting autonomic modulation could influence this imbalance in the study population. The primary objective of this study was to evaluate the immediate and short-term effects of VMT on ANS modulation, as measured by HRV, in individuals with probable bruxism. The secondary objective was to assess the effects of VMT on the viscoelastic properties and pressure pain sensitivity of the masticatory muscles, given that bruxism potentially contributes to muscle overload and increases the risk of temporomandibular disorders (TMDs). The null hypothesis states that there will be no statistically significant differences between the intervention and control groups in any of the outcome measures.

## 2. Materials and Methods

### 2.1. Study Design

An experimental, parallel-group, single-blind, randomized controlled trial was conducted. The study adhered to the CONSORT 2025 statement for reporting clinical trials [28] and complied with the ethical principles of the Declaration of Helsinki. The research protocol was approved by the Institutional Ethics Committee (approval code: 1818-N-22, approval date: 25 January 2023) and was prospectively registered at ClinicalTrials.gov (registration code: NCT05751694, registration date: 2 March 2023).

### 2.2. Participants

Using a convenience sampling method, consecutive individuals diagnosed with bruxism by a dentist and referred for physiotherapy were recruited from November 2023 to January 2025 at a private medical center in Southern Spain. Eligible participants were aged between 18 and 65 years and met the criteria for “probable bruxism” according to the diagnostic guidelines proposed by Lobbezoo et al. [2]. The diagnosis requires a positive self-report combined with the presence of one or more clinical signs, including (a) visible tooth wear, chipping, or fractures; (b) dental impressions on the tongue and/or cheeks; (c) hypertrophy of the masseter muscles; and (d) tenderness upon palpation of the masticatory muscles [2]. Exclusion criteria included (a) recent trauma or fractures in the craniofacial, mandibular, or cervical regions; (b) previous temporomandibular joint surgery; (c) acute dental pain due to caries or root inflammation; (d) ongoing orthodontic treatment; (e) history of abdominal surgery, gastric ulcers, gastritis, or current/past gastric neoplasia; (f) diagnosis of neurological, rheumatic, or systemic diseases; (g) pregnancy or breastfeeding; (h) current chemotherapy or radiotherapy treatment; (i) cognitive or psychiatric disorders; (j) cardiac conditions, including arrhythmias or the presence of implanted electronic devices; (k) drug or alcohol abuse; (l) use of analgesics or drugs affecting the central nervous system; and (m) prior experience with VMT directed to the gastric region. All participants received both detailed oral and written information about the study and signed an informed consent form before being enrolled.

### 2.3. Randomization and Blinding

An external staff member used Microsoft Excel to generate a random number sequence with a 1:1 allocation ratio. Group assignment was concealed using opaque, sealed, and consecutively numbered envelopes. The outcome assessor remained blinded to participants’ group allocation.

### 2.4. Interventions

**Figure 1:**
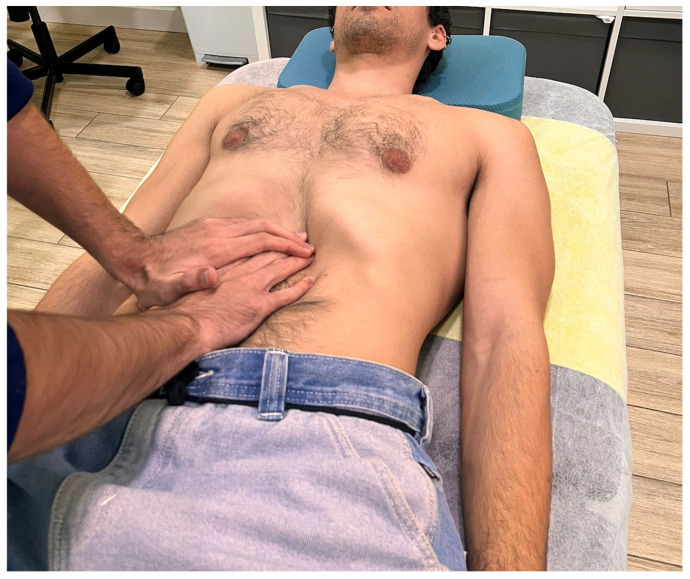
Hand placement during the first stage of the visceral manual therapy technique.

A physiotherapist with over 10 years of clinical experience in MT was responsible for the interventions across both study groups. Interventions took place in a private physiotherapy clinic, where separate rooms were used for evaluation and treatment to ensure blinding and consistency. A treatment table and a bolster were used during sessions. The gastric region was selected as the target area for the VMT technique due to its rich vagal innervation and its direct anatomical connections with key brainstem nuclei, including the spinal trigeminal nucleus. A trigeminovagal interface has been described at this level, providing a convergence site between visceral and craniofacial afferences and a strategic target to modulate parasympathetic activity through vagal stimulation [29]. The experimental group received a VMT technique adapted from the “Ralph Failor Osteopathic Technique” [30]. Participants were placed in a supine position with hips, knees, and ankles flexed, and a bolster was placed under the knees to relax the abdominal region. The technique was administered in two sequential stages. In the first stage, the therapist placed both hands over the epigastric region, just below the xiphoid process, and applied progressive pressure towards the upper border of the stomach’s greater curvature. A skin fold was lifted to reduce superficial tension and direct contact to the underlying visceral tissue (Figure 1).

**Figure 2:**
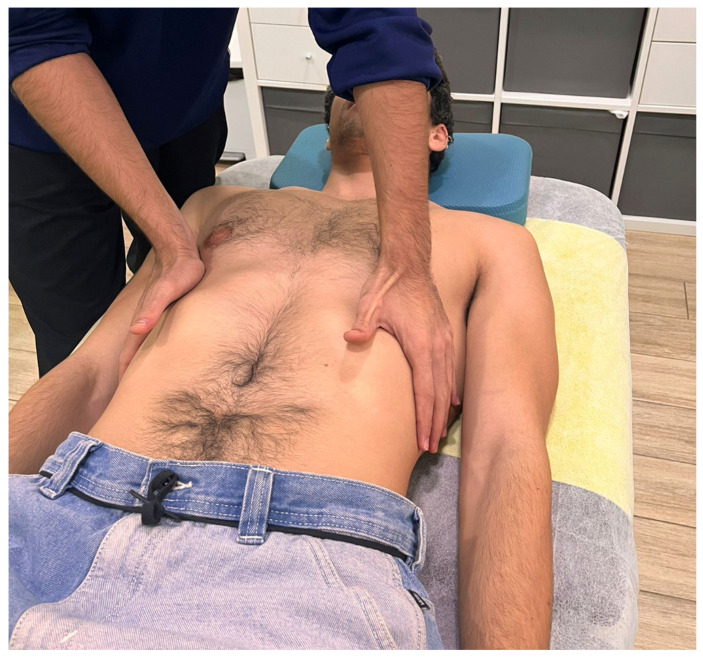
Hand placement during the placebo technique.

Participants were instructed to breathe deeply throughout the intervention. During exhalation, the therapist applied a zigzag motion with vibratory hand movements while exerting caudal traction on the underlying tissues. The whole procedure lasted approximately 3 min. In the second stage, the therapist’s left hand was positioned over the epigastric area, with the ulnar border placed under the inferior edge of the left costal margin (Figure 2). The right hand was used to reinforce the contact. Once again, the participant was instructed to breathe deeply, and during exhalation, the therapist applied sustained caudal traction for approximately 2 min.

**Figure 3:**
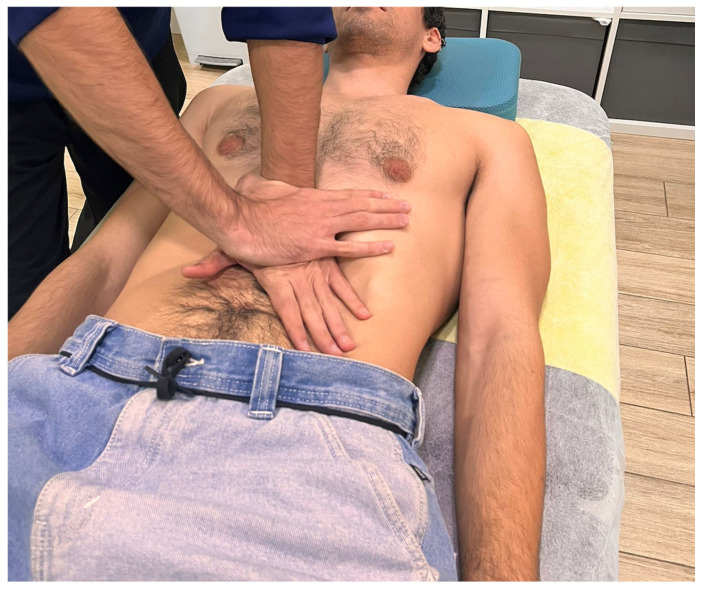
Hand placement during the second stage of the visceral manual therapy technique.

To ensure an effective control condition, the sham intervention was carefully designed to mimic the contextual and sensory aspects of the experimental procedure while omitting the therapeutic mechanical stimuli inherent to VMT. Specifically, no pressure, tension, or traction was applied to the soft tissues, in line with prior sham MT protocols [25,26,31]. The therapist placed both hands at the lower costal margin (Figure 3) and instructed the patient to breathe deeply, as in the experimental group. Gentle static contact was maintained on this region, without inducing any tissue mobilization or engagement. This approach aimed to control for non-specific effects, including attentional focus, relaxation, and placebo responses, enabling a more accurate attribution of the observed effects to the specific physiological impact of the active intervention, therefore potentially enhancing the internal validity of the study. In both groups, participants underwent two treatment sessions with a 1-week interval between them.

### 2.5. Outcome Measures

An experienced physiotherapist who remained blinded to the study aims and participants’ group allocation was responsible for all assessments. Outcome measures were collected at baseline (T1), immediately after the first treatment session (T2), before the second intervention at week 2 (T3), immediately after the second intervention at week 2 (T4), and at a 4-week follow-up (T5) [25].

#### 2.5.1. Primary Measure—HRV

HRV was measured using a Polar H10 chest strap (Polar Electro Oy., Kempele, Finland) [32,33] and the Elite HRV smartphone app (Elite HRV Inc., Asheville, NC, USA) [34,35]. Participants were placed in a supine position with the head supported on a pillow. After a 5 min resting period, HRV was recorded continuously for 5 min. The collected data were then transferred and analyzed using Kubios HRV Standard 3.5.1 software (Kubios Oy., Kuopio, Finland) [36]. Two HRV parameters were extracted for analysis, namely the root mean square of the successive differences (HRV-RMSSD) and standard deviation of the normal-to-normal interbeat intervals (HRV-SDNN) [37]. HRV-RMSSD reflects vagal activity [38], while HRV-SDNN assesses overall HRV as a measure of the balance between sympathetic and parasympathetic activity [39].

#### 2.5.2. Secondary Measures

Pressure pain sensitivity was evaluated using a digital algometer (Wagner Force Ten™, model FPX 100, Wagner Instruments, Riverside, CN, USA) to determine pressure pain thresholds (PPTs) as the minimum necessary pressure to evoke pain or discomfort. PPTs were measured at the spinous process of C4 and bilaterally at both the masseter and temporalis muscles [40]. For the evaluation of the masticatory muscles, the patient was placed in a side-lying position, contralateral to the side being evaluated, with the head on a pillow and the lower limbs flexed. The measurement point for the masseter muscle was located 1 cm superior and 2 cm anterior to the mandibular angle, and the temporalis muscle point was located over the anterior fibers of the temporalis muscle, approximately 2 cm above the zygomatic arch, midway between the lateral edge of the eye and the anterior portion of the helix [41]. For C4 assessment, participants were seated upright, and perpendicular pressure was applied to the spinous process. At all locations, the assessor gradually increased the pressure and instructed the individuals to indicate when the pressure began to feel unpleasant or painful [25]. Two measurements were taken, with a 30 s interval between them, and the average of the two was used for further analysis.

Muscle viscoelastic properties were assessed using the Myoton PRO device (MYOTON AS, Tallinn, Estonia) [42]. Two parameters were evaluated [43], namely the natural oscillation frequency (F), which reflects the muscle tone or state of tension; and dynamic stiffness (S), which represents the muscle resistance to an external force or deformation. Measurements were conducted in the same contralateral side-lying position as for PPTs, using the same anatomical landmark. Two measurements were taken, with a 30 s interval rest, and the average of the two was recorded for subsequent analysis.

### 2.6. Sample Size

The sample size was estimated for a two-group design with five repeated measurements. Parameters included an alpha level of 0.05, a study power of 95%, a correlation coefficient of 0.4 between repeated measures, and a medium effect size (η^2^ ≈ 0.12) for differences between groups in HRV, as assessed with the HRV-SDNN (G*Power software, version 3.1.9.7, University of Kiel, Kiel, Germany). To account for a potential 10% dropout rate, a total of 24 participants were required to complete the study.

### 2.7. Statistical Analysis

Statistical analyses were performed using IBM Statistics Package for Social Science (SPSS^®^), version 29 (IBM Corp., New York, NY, USA), following an intention-to-treat principle. The assumption of normality was tested for all continuous variables using the Shapiro–Wilk test. Based on this, parametric or non-parametric tests were selected accordingly. Between-group differences at each time point were examined using the Mann–Whitney U test. To analyze the effects of the intervention over time, a repeated-measures analysis of variance (ANOVA) was conducted using the group (experimental vs. control) as the between-subjects factor and time (baseline [T1], immediately after the first session [T2], before the second session [T3], immediately after the second session [T4], and 4-week follow-up [T5]) as the within-subjects factor. The effect size was estimated using partial eta squared (η^2^). The level of significance was set at *p* value < 0.05.

## 3. Results

**Figure 4:**
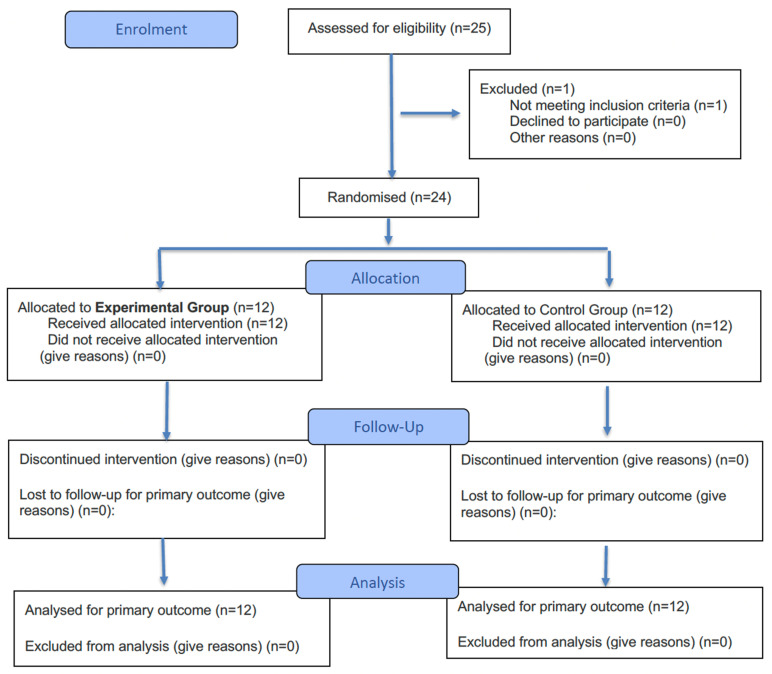
Flowchart diagram of participants.

A total of 24 participants (11 females, 45.83%) with a mean age of 38 ± 10.60 years and a clinical diagnosis of probable bruxism were included in the study. No participants were lost to follow-up throughout the study period (Figure 4). There were no modifications to the intervention protocol, and no adverse effects were reported during the course of the study. Table 1 lists the clinical and demographic characteristics of the sample. At baseline, there were no statistically significant differences between groups for any of the study measures (all, *p* > 0.05).

### 3.1. Primary Outcome—HRV

The ANOVA showed a significant time × group interaction for HRV-SDNN, with a medium effect size (F = 2.90; *p* = 0.04; η^2^ = 0.12), but not for HRV-RMSSD. Post hoc pairwise comparisons revealed significant differences between groups with large effect sizes when comparing T1 to T2 for both HRV-SDNN and HRV-RMSSD. Similarly, significant changes were observed when comparing T3 to T4 (Table 2).

### 3.2. Secondary Outcomes

No significant time×group interaction was observed for PPT scores or the viscoelastic properties of the masseter muscle (all, *p* > 0.05) (Supplementary Materials Tables S1 and S2). Post hoc pairwise comparisons only showed a significant increase in the PPT at C4 when comparing T3 and T4 (*p* = 0.049), with a large effect size (η^2^ = 0.16; 95% CI: 0.00–0.40), and in the left temporalis muscle when comparing T1 and T2 (*p* = 0.01), with a medium effect size (η^2^ = 0.07; 95% CI: 0.00–0.32).

## 4. Discussion

The present randomized controlled trial investigated the immediate and short-term effects of VMT on ANS activity in individuals diagnosed with probable bruxism and whether these effects were accompanied by changes in the viscoelastic properties and pressure pain sensitivity of the masticatory muscles. The results support the rejection of the null hypothesis regarding autonomic modulation, as significant changes were observed for HRV. However, the null hypothesis could not be rejected for muscle tone, stiffness, or pressure pain sensitivity, as no consistent significant changes or differences between groups were observed. Our findings demonstrated a significant increase in HRV-SDNN over time in those who underwent VMT compared to the sham placebo group, which suggests that VMT may positively modulate sympathovagal balance. Conversely, VMT was no more effective than the placebo at eliciting changes in muscle viscoelastic properties and the PPTs of the masticatory muscles. The sham intervention was designed to replicate contextual and sensory aspects of the experimental procedure without providing a specific mechanical input and, therefore, control for factors such as therapist interaction, physical contact, and patient expectations, which are known to influence the perceived benefits following MT [44]. Since these non-specific aspects may contribute to the overall therapeutic outcome, using a sham intervention helps to strengthen the internal validity of the study by isolating the specific effects attributed to the VMT technique. However, this approach may also reduce the magnitude of the observed differences between groups due to a plausible placebo effect, which needs to be considered when interpreting the results.

Previous evidence has concluded that MT applied to the diaphragm may elicit immediate changes in HRV in patients with respiratory conditions [45] and that VMT targeting the epigastric region may help to regulate the lower esophageal sphincter tone in individuals with gastroesophageal reflux disease [25,26]. However, to our knowledge, no prior research has directly assessed the impact of VMT on ANS function. While the present preliminary findings are promising and may support the potential role of VMT to modulate autonomic function in individuals with bruxism, these results should be interpreted cautiously and require confirmation through larger trials before establishing clinical recommendations. It is important to emphasize that improving autonomic modulation may not directly affect bruxism behaviors, but it may potentially influence signs and symptoms related to temporomandibular problems, which are sometimes present in individuals with bruxism.

Although our study demonstrated significant improvements in HRV-SDNN and HRV-RMSSD following VMT, the clinical relevance of these changes in individuals with bruxism remains to be fully established. HRV is recognized as a global indicator of patient health and should be interpreted within a broader clinical context, given its sensitivity to multiple factors and conditions, such as chronic stress, circadian rhythm disturbances, metabolic syndrome, and a sedentary lifestyle, among others. Moreover, HRV can serve as an objective marker to monitor clinical improvements in different health conditions [15]. Future research should explore the relationship between HRV modulation and functional or clinical outcomes, including sleep quality, clenching activity, and perceived stress levels, to better understand the significance of these responses in people with bruxism.

Contrary to what was expected, changes in pressure pain sensitivity were minimal and only achieved statistical significance in post hoc pairwise comparisons in the C4 spinous process and the left temporalis muscle. McCross et al. [46] concluded that MT applied to the epigastric region may evoke a neuromodulatory response via afferent stimulation of the phrenic nerve, a mechanism described as “regional interdependent inhibition”. Similarly, it has been hypothesized that VMT could induce hypoalgesic responses through vagal activation and the trigeminovagal convergence at the level of the spinal trigeminal nucleus [29]. Despite these purported theoretical mechanisms, the present results do not fully support this hypothesis and provide only limited empirical rationale for such effects. Previous studies have demonstrated that MT can reduce the pressure pain sensitivity of myofascial trigger points in patients with TMDs [47], which, although distinct from bruxism, may share overlapping clinical consequences, such as masticatory muscle overload. Our findings should be interpreted within this context, as VMT is not intended to directly modify bruxism behavior. Studies specifically focused on individuals with bruxism are scarce. In line with our findings, Machado et al. [48] reported no significant changes in PPTs of the masticatory muscles in a follow-up study in adults with bruxism undergoing orthodontic treatment. In contrast, Kadıoğlu et al. (2024) [23] found that an 8-week intervention combining MT (including stretching, mobilizations, and ischemic compression) with a home exercise program reduced pain intensity in patients with bruxism presenting symptoms consistent with TMDs due to muscular overload. The protocol of this study also involved the activation of the parasympathetic nervous system through stretching of the suboccipital muscles and cervical fascia. Differences in the intervention protocols between studies may explain the discrepancies with our results.

Regarding the viscoelastic properties of masticatory muscles, no significant changes were observed in muscle tone or stiffness of the masticatory muscles. Microtraumas induced by bruxism are among the contributing factors to the development of TMDs [49]. Although patients with mild TMDs show similar masticatory muscle stiffness than those without dysfunction [43], individuals diagnosed with a moderate-to-severe TMD exhibit significant changes across all myotonometry parameters, including increased muscle tone and stiffness of the masseter muscles compared to healthy individuals, with differences ranging from 12.5% to 16.5% [50]. Similarly, women with muscular or mixed (muscular and articular) TMDs show altered viscoelastic properties of the masticatory muscles when compared to asymptomatic controls [51]. The discrepancy between these findings and our results may be due to differences in the study sample. Participants in this clinical trial were referred by dentists with a diagnosis of “probable bruxism”, and the presence or absence of a TMD diagnosis was not assessed. This distinction is important, as bruxism can be present without muscle symptoms, whereas TMDs are characterized by muscle pain, which was not the main focus of our study.

### 4.1. Study Limitations

Several limitations should be acknowledged when interpreting the present results. First, although the sample size was deemed appropriate based on the study aims and was previously estimated, it remains relatively small. This limits the generalizability of the findings and increases the risk of type II errors, particularly for outcomes with a small effect size. Nevertheless, statistically significant differences were observed in key outcomes, and the effects sizes can be considered robust. Given the exploratory nature of the study, these findings should be interpreted cautiously and warrant further confirmation. Second, the use of convenience sampling based on dental referrals may introduce selection bias, as the sample may not fully represent the general population of individuals with bruxism. Future studies should aim for more diverse recruitment strategies to enhance external validity. Third, the short follow-up period (4 weeks) did not allow for the evaluation of long-term effects. Future studies should consider longer follow-up periods. Fourth, the diagnosis of probable bruxism was based on self-reporting and clinical evaluation by a dentist, which may reduce diagnostic accuracy. This may have influenced the observed results, particularly the absence of significant changes in PPTs and myotonometry scores. It would be of interest to incorporate valid diagnostic tools, such as the Standardised Tool for the Assessment of Bruxism (STAB) and objective classification criteria to enhance comparability across studies and strengthen the clinical research in this field.

### 4.2. Possible Applications of Research and Future Research Directions

Our findings suggest that two sessions of VMT may lead to short-term modulation of the ANS, as reflected by changes in HRV. However, the clinical applicability of the results remains to be established. Future research should explore the role of VMT as part of a comprehensive multimodal approach for individuals with bruxism. Although VMT may exert a neuromodulatory effect on the ANS, as assessed with HRV-SDNN and HRV-RMSSD, this effect did not result in measurable changes in muscle tone, stiffness, or pressure pain sensitivity. This may support the notion that autonomic responses and local musculoskeletal outcomes may involve different physiological mechanisms and temporal trajectories. Further research is warranted to better understand the relationship between autonomic modulation and musculoskeletal responses.

Given that the ANS is also involved in emotional regulation, stress responses, and overall homeostasis, future research should incorporate Axis II characterization to provide a more comprehensive understanding of the biopsychosocial profile of adults with bruxism. Investigating correlations between physiological changes and functional, subjective, and psychosocial outcomes would enhance our understanding of the clinical relevance of autonomic modulation. Additionally, the use of valid diagnostic tools is strongly recommended.

## 5. Conclusions

The study findings suggest that two sessions of VMT may lead to a short-term modulation of ANS activity, as reflected by changes in HRV. However, VMT did not demonstrate better effects than the placebo in modifying pressure pain sensitivity or the viscoelastic properties of the masticatory muscles. Further research involving larger sample sizes, longer follow-up periods, and comprehensive diagnostic criteria is warranted to determine the clinical relevance and applicability of VMT to manage mandibular-related signs and symptoms in individuals with bruxism.

## Figures and Tables

**Table 1:** Baseline demographic characteristics of participants in the study groups.

	Experimental Group n = 12	Control Group n = 12
Age, y, mean (SD) [Range]	38.5 (9.64) [21–55]	38.5 (11.92) [25–64]
Sex, female, n (%)	5 (41.66%)	6 (50%)
Height, cm, mean (SD) [Range]	168.25 (8.42) [154–178]	170.00 (10.89) [156–187]
Weight, Kg, mean (SD) [Range]	75.89 (19.42) [46.30–104.10]	89.97 (22.27) [55–133]
BMI, Kg/cm^2^, mean (SD) [Range]	26.40 (4.67) [19.52–33.23]	30.96 (6.55) [22.60–43.96]

BMI (body mass index), SD (standard deviation).

**Table 2:** Changes in heart rate variability over time.

	Within-Group Differences		Between-Group Differences	Effect Size
	Experimental Group	Control Group		
**HRV–SDNN**				
Baseline (T1)	39.64 (12.01)	38.76 (23.26)		
Post-first intervention (T2)	58.25 (24.00)	38.34 (21.79)		
Pre-second intervention (T3)	41.21 (21.14)	32.50 (9.97)		
Post-second intervention (T4)	48.21 (14.75)	32.51 (15.49)		
4-week follow-up (T5)	41.21 (21.14)	32.50 (9.97)		
Change T1 to T2	−18.61 (15.96)	0.42 (4.23)	**0.001 (−15.43 to −2.76)**	0.42 (0.10–0.62)
Change T3 to T4	−11.61 (18.16)	−3.42 (8.58)	0.172 (−13.64 to −1.39)	0.08 (0.00–0.32)
**HRV–RMSSD**				
Baseline (T1)	36.15 (14.73)	40.63 (36.37)		
Post-first intervention (T2)	54.41 (25.86)	40.59 (35.91)		
Pre-second intervention (T3)	33.56 (8.46)	28.15 (14.60)		
Post-second intervention (T4)	45.93 (16.20)	31.30 (19.16)		
4-week follow-up (T5)	39.74 (24.00)	34.09 (17.65)		
Change T1 to T2	−18.26 (−29.02 to −7.49)	0.04 (−2.03 to 2.10)	**0.001 (−15.51 to −2.70)**	0.38 (0.07–0.59)
Change T3 to T4	−12.37 (−22.46 to −2.29)	−3.15 (−7.80 to 1.50)	0.08 (−13.24 to −2.28)	0.13 (0.00- 0.38)

Data are reported as mean (standard deviation) or mean (95% confidence interval). Bold data indicates statistical significance (95% CI does not cross zero; *p*-value < 0.05). HRV—SDNN (heart rate variability—standard deviation of the normal-to-normal interbeat intervals); HRV—RMSSD (heart rate variability—root mean square of the successive differences).

## Data Availability

The data presented in this study is available through the institutional open access repository https://doi.org/10.12795/11441/174370 (accessed on 22 June 2025).

## Supplementary Materials

The following supporting information can be downloaded at: https://www.mdpi.com/article/10.3390/dj13070325/s1, Table S1: Pressure pain threshold scores: mean values over time and comparison of variations between consecutive time points. Table S2: Muscle viscoelastic properties of the masseter muscle (muscle tone, F; stiffness, S): mean values over time and comparison between consecutive time points.

## Abbreviations

The following abbreviations are used in this manuscript:

ANS	Autonomic nervous system
VMT	Visceral manual therapy
HRV	Heart rate variability
TMD	Temporomandibular disorder
HRV-RMSSD	Root mean square of the successive differences
HRV-SDNN	Standard deviation of the normal-to-normal interbeat intervals
PPT	Pressure pain threshold
MT	Manual therapy
ANOVA	Analysis of variance
