# Supplementary material for: Short-Term Effects of Visceral Manual Therapy on Autonomic Nervous System Modulation in Individuals with Clinically Based Bruxism: A Randomized Controlled Trial
Source: Dent J (Basel). 2025 Jul 16;13(7):325. doi: 10.3390/dj13070325 (PMC12293791; doi:10.3390/dj13070325)
Supplement: Supplementary file 1 [file dentistry-13-00325-s001.zip › dentistry-3644151-supplementary.pdf]

Table S1 – Pressure pain threshold scores: mean values over time and comparison of variations between consecutive time points.

	Within group diffrences		Between group differences
	Experimental Group	Control Group	
**C4**			
Baseline (T1)	2.93 (1.13)	2.90 (1.38)	
Post- first intervention (T2)	2.82 (1.07)	2.71 (1.01)	
Pre- second intervention (T3)	3.05 (1.35)	2.84 (0.97)	
Post- second intervention (T4)	3.52 (1.62)	2.92 (1.02)	
4-week follow-up (T5)	3.42 (1.46)	3.19 (1.02)	
Change T1 to T2	0.11 (-0.28 to 0.51)	0.19 (-0.16 to 0.55)	0.74 (-0.09 to 0.40)
Change T3 to T4	-0.47 (-0.81 to -0.13)	-0.08 (-0.32 to 0.16)	**0.049** (-0.48 to -0.07)
**Right Masseter**			
Baseline (T1)	1.71 (0.71)	1.90 (0.93)	
Post- first intervention (T2)	1.84 (0.73)	1.92 (0.74)	
Pre- second intervention (T3)	2.08 (0.74)	1.88 (0.72)	
Post- second intervention (T4)	2.15 (0.72)	1.98 (0.81)	
4-week follow-up (T5)	2.16 (0.78)	2.10 (0.83)	
Change T1 to T2	-0.13 (-0.25 to -0.002)	-0.02 (-0.21 to 0.17)	0.30 (-0.21 to 0.04)
Change T3 to T4	-0.07 (-0.24 to 0.10)	-0.10 (-0.32 to 0.12)	0.80 (-0.21 to 0.04)
**Left Masseter**			
Baseline (T1)	1.64 (0.57)	1.82 (0.56)	
Post- first intervention (T2)	1.81 (0.73)	1.84 (0.63)	
Pre- second intervention (T3)	2.09 (0.86)	1.82 (0.67)	
Post- second intervention (T4)	2.20 (0.77)	1.79 (0.68)	
4-week follow-up (T5)	2.17 (0.78)	1.99 (0.69)	
Change T1 to T2	-0.17 (-0.33 to -0.23)	-0.02 (-0.22 to 0.18)	0.18 (-0.17 to 0.09)
Change T3 to T4	-0.11 (-0.38 to 0.15)	0.02 (-0.06 to 0.11)	0.283 (-0.18 to 0.09)
**Right Temporalis**			
Baseline (T1)	2.10 (0.78)	2.18 (0.69)	
Post- first intervention (T2)	2.32 (1.03)	2.32 (0.57)	
Pre- second intervention (T3)	2.56 (1.26)	2.14 (0.50)	
Post- second intervention (T4)	2.76 (1.20)	2.13 (0.65)	
4-week follow-up (T5)	2.62 (1.02)	2.38 (0.66)	
Change T1 to T2	-0.22 (-0.55 to 0.11)	-0.13 (-0.31 to 0.04)	0.63 (-0.35 to -0.006)
Change T3 to T4	-0.21 (-0.50 to 0.08)	0.01 (-0.20 to 0.22)	0.19 (-0.27 to 0.07)
**Left Temporalis**			
Baseline (T1)	2.19 (0.98)	2.45 (0.83)	
Post- first intervention (T2)	1.81 (0.73)	1.84 (0.63)	
Pre- second intervention (T3)	2.61 (1.21)	2.14 (0.55)	
Post- second intervention (T4)	2.78 (1.28)	2.24 (0.64)	
4-week follow-up (T5)	2.64 (1.25)	2.40 (0.81)	
Change T1 to T2	-0.27 (-0.5 to 0.005)	0.15 (-0.06 to 0.006)	**0.01** (-0.24 to 0.12)
Change T3 to T4	-0.17 (-0.30 to 0.03)	-0.10 (-0.21 to 0.01)	0.68 (-0.30 to 0.03)

Data are reported as mean (standard deviation) or mean (95% confidence interval). Bold data indicates statistical significance (95% CI does not cross zero; p-value < 0.05).

Table S2 – Muscle viscoelastic properties of the masseter muscle (muscle tone, F; and Stiffness, S): mean values over time and comparison between consecutive time points.

	Within group differences		Between group differences
	VMT Group	Control Group	
**Right Masseter (tone)**			
Baseline (T1)	18.94 (5.21)	19.38 (4.38)	
Post- first intervention (T2)	18.99 (5.37)	17.66 (3.90)	
Pre- second intervention (T3)	19.05 (5.53)	17.98 (3.04)	
Post- second intervention (T4)	18.58 (5.30)	17.68 (3.39)	
4-week follow-up (T5)	19.05 (4.84)	19.58 (6.18)	
Change T1 to T2	-0.04 (-0.86 to 0.78)	1.72 (-0.18 to 3.61)	0.07 (-0.18 to 1.86)
Change T3 to T4	0.47 (-0.04 to 0.98)	0.30 (-0.55 to 1.15)	0.70 (-0.07 to 0.84)
**Left Masseter (tone)**			
Baseline (T1)	18.84 (5.799	17.84 (3.45)	
Post- first intervention (T2)	18.58 (5.69)	18.76 (4.43)	
Pre- second intervention (T3)	20.19 (6.39)	18.24 (3.62)	
Post- second intervention (T4)	19.61 (6.12)	16.95 (4.18)	
4-week follow-up (T5)	19.21 (4.79)	18.64 (3.12)	
Change T1 to T2	0.26 (-0.81 to 1.35)	-0.92 (-2.4 to 0.53)	0.164 (-1.20 to 0.54)
Change T3 to T4	0.57 (-0.45 to 1.60)	1.29 (-0.31 to 2.88)	0.418 (0.04 to 1.81)
**Right Masseter (stiffness)**			
Baseline (T1)	433.46 (138.86)	441.96 (130.99)	
Post- first intervention (T2)	433.42 (130.64)	393.67 (104.26)	
Pre- second intervention (T3)	447.25 (140.53)	395.04 (70.46)	
Post- second intervention (T4)	425.54 (124.40)	402.75 (93.89)	
4-week follow-up (T5)	428.20 (135.70)	408.87 (76.61)	
Change T1 to T2	0.04 (-28.80 to 28.88)	48.29 (-11.07 to 107.65)	0.12 (-7.90 to 56.23)
Change T3 to T4	21.71 (-2.12 to 45.54)	-7.71 (-28.92 to13.50)	0.055 (-8.98 to 22.98)
**Left Masseter (stiffness)**			
Baseline (T1)	419.33 (143.94)	397.42 (92.44)	
Post- first intervention (T2)	403.83 (133.73)	412.71 (115.38)	
Pre- second intervention (T3)	452.50 (144.63)	399.58 (89.95)	
Post- second intervention (T4)	433.62 (158.80)	391.92 (86.65)	
4-week follow-up (T5)	426.50 (117.44)	415.21 (80.78)	
Change T1 to T2	15.50 (-20.02 to 51.02)	-15.29 (-59.30 to 28.71)	0.24 (-26.72 to 26.93)
Change T3 to T4	18.88 (-18.94 to 56.70)	7.67 (-11.96 to 27.29)	0.57 (-6.46 to 33.01)

Data are reported as mean (standard deviation) or mean (95% confidence interval). Bold data indicates statistical significance (95% CI does not cross zero; p-value < 0.05).
